# Fault Detection and Diagnosis in Industrial Processes with Variational Autoencoder: A Comprehensive Study

**DOI:** 10.3390/s22010227

**Published:** 2021-12-29

**Authors:** Jinlin Zhu, Muyun Jiang, Zhong Liu

**Affiliations:** 1 State Key Laboratory of Food Science and Technology, Jiangnan University, Wuxi 214122, China; 2 School of Food Science and Technology, Jiangnan University, Wuxi 214122, China; 3 School of Computer Science and Engineering, Nanyang Technological University, Singapore 639798, Singapore; james.jiang@ntu.edu.sg; 4 Key Laboratory of Advanced Process Control for Light Industry (Ministry of Education), Jiangnan University, Wuxi 214122, China; 6211924088@stu.jiangnan.edu.cn

**Keywords:** process monitoring, deep model, variational autoencoder, deep reconstruction, dynamic process

## Abstract

This work considers industrial process monitoring using a variational autoencoder (VAE). As a powerful deep generative model, the variational autoencoder and its variants have become popular for process monitoring. However, its monitoring ability, especially its fault diagnosis ability, has not been well investigated. In this paper, the process modeling and monitoring capabilities of several VAE variants are comprehensively studied. First, fault detection schemes are defined in three distinct ways, considering latent, residual, and the combined domains. Afterwards, to conduct the fault diagnosis, we first define the deep contribution plot, and then a deep reconstruction-based contribution diagram is proposed for deep domains under the fault propagation mechanism. In a case study, the performance of the process monitoring capability of four deep VAE models, namely, the static VAE model, the dynamic VAE model, and the recurrent VAE models (LSTM-VAE and GRU-VAE), has been comparatively evaluated on the industrial benchmark Tennessee Eastman process. Results show that recurrent VAEs with a deep reconstruction-based diagnosis mechanism are recommended for industrial process monitoring tasks.

## 1. Introduction

Statistical process monitoring (SPM) is an important decision-making module in modern manufacturing sectors, allowing them to achieve higher plant safety, product quality, and enterprise profitability [1]. Currently, the ongoing Industry 4.0 movement has also brought new thrust and opportunities to SPM, due to key enablers such as the pervasive sensory module deployment, unprecedented Internet of things (IoT) connection and communication, low-cost massive data storage, as well as the ever-increasingly powerful computation technologies. Thus, to distill advisable knowledge and intelligence from real-time process data and to promote timely decision making, the study of SPM, including data-driven process modeling, fault detection, and fault diagnosis, have been highly focused among the smart manufacturing community [2].

Traditional statistical process analysis is notably dominated by principal component analysis (PCA). As the pioneering milestone for coping with high-dimensional and correlated process data, PCA performs the feature extraction by transforming the original process variables and yields on an orthogonal basis, in which different dimensions become uncorrelated [3]. Those basis vectors are called principal components. By looking into the latent projections which cover the most informative viewpoint, two monitoring statistics, called Hoteling’s T2 and squared prediction error (SPE), are commonly constructed. Hoteling’s T2 monitors the unexpected variations in latent space while the SPE inspects the residual space. One can also build up a single index by combining the two indexes through proper weighting [4]. In this way, rather than processing two indices, only one combined index is utilized for process monitoring. For fault diagnosis, the contribution plots and reconstruction-based methods are generally utilized [5]. With this well-formulated prototype, extensions can be found to address industrial nonlinearity and/or time-wise correlations. For instance, the kernel PCA uses the kernel transformation so that PCA can be performed in a reproducing kernel Hilbert space [6]. The work by [7] considers nonlinear PCA (NLPCA) modeling by using a five-layer auto-associative neural network. To deal with the time-dependence, the dynamic PCA has been suggested as a remedy by adding time-lagged observations [8]. In [9], the authors proposed a dynamic latent variable modeling algorithm, and an auto-regressive mechanism has been embedded to carve out dynamics in latent vectors. Recently, one can also find several other improvements, such as a hybrid framework to automate fault detection and diagnosis that is based on moving window principal component analysis (MWPCA) and Bayesian networks (BN) [10], a fractal-based DKPCA (FDKPCA) [11], and a two-step localized KPCA (TSLKPCA) [12].

Despite the great success of the PCA methods, some issues may still arise in present-day industrial process modeling and monitoring. First of all, consider the computation cost of modeling voluminous data set; the caveat with KPCA is that a large data set can always lead to a large kernel matrix for computation and storage, while for DPCA, the innate issue is similar, due to the augmented data matrices. Second, as an essentially shallow model, the effectiveness of PCA on feature extraction and knowledge representation can be rather limited for decision making. Third, the monitoring of some methods are only intended for fault detection; fault diagnosis has not been well formulated. Recently, as a promising alternative to this dilemma, the deep learning-based monitoring strategy has been embraced. A deep neural network (DNN) is commonly designed with multiple layers between the input and output layers. In this way, top layers enable the abstract composition of features from lower layers, through which those task irrelevant features will be down-clamped and informative features will be better organized [13]. As a result, deep networks can be used to model highly nonlinear and dynamic objects and have made remarkable achievements in a wide range of industrial applications, including natural language processing (NLP) [14], social network studies, and biology system longitudinal analyses [15]. For fault detection and diagnosis, a new deep neural network, the multichannel one-dimensional convolutional neural network (MC1-DCNN), is proposed to investigate feature learning from high-dimensional process signals from the literature [16].

For the relevant research in process monitoring, one can find that autoencoders (AEs) and variational autoencoders (VAEs), as the two primitive deep models, have been recently applied in industrial systems. In the literature, [17] proposed the dynamic stacked auto-encoder model to extract discriminative features for fault classification. For fault detection, variant autoencoders such as denoising autoencoders and contractive autoencoders have been evaluated in extracting nonlinear feature representations for the fault detection of industrial processes in [18]; the results show that both models can deliver simple and effective performance. Technically, the VAE is the generalization of AEs, with regularization to avoid overfitting and also to ensure that the latent space has good generative properties. Recently, the variational autoencoder has been successfully developed for nonlinear process monitoring [19,20]. To further consider the temporal relations, in [21], a variational recurrent autoencoder has been built, which takes both nonlinearities and dynamics into account. To obtain the characteristics of an informational manifold with raw data, an adversarial autoencoder is proposed in [22]. Recently, a new fault detection method, a convolutional gated recurrent unit auto-encoder (CGRU-AE), for feature learning from process signals is proposed in [23]. All these works have recognized that deep generative models can often outperform shallow generative models in process monitoring tasks.

Despite the impressive merits of intelligent monitoring, one should still note that, compared to the well-disposed PCA, the fault detection and diagnosis capabilities of VAE and its variants have not been well formulated and investigated. In view of this, this work aims to provide a systematic monitoring flowchart and a comprehensive study for deep VAE models. The contribution can be summarized with the following three aspects. First, the fault detection schemes for deep models have been studied and discussed under three different diagrams. Second, we propose two deep learning-based diagnosis methods, namely, the deep contribution plot (dCP) and the deep reconstruction-based contribution (dRBC) plot, for deep fault diagnosis. Third, the fault detection and diagnosis capabilities have been established for the VAE variants, and then all deep monitoring paradigms are comparatively studied on the TE process.

The rest of the paper is organized as follows. Section 2 gives the fundamental modeling theory with the VAE variants. Then, in Section 3, we define the fault detection and fault diagnosis mechanisms. A case study is conducted in Section 4. The last section features our conclusions.

## 2. Process Modeling with VAE

Given the process data, $X={\left\{x_{k}\in R^{D}\right\}}_{k=1}^{N}$ from N observations. This section will revisit three VAE variants for process modeling, namely, the static VAE model, the dynamic VAE model, and the recurrent VAE model.

### 2.1. Static VAE

The static VAE is a probabilistic generative model based on a neural network. Suppose there exists a latent variable z that generates the observation variable x, $z\in R^{d}$, d<D; the goal is to determine a posterior distribution of the latent variable with Bayes’ rule:

(1) $$p\left(z|x\right)=\frac{p\left(x|z\right)p\left(z\right)}{p\left(x\right)}.$$

However, the above denominator, p(x)=∫p(x|z)p(z)dz, is intractable due to the high dimension integral. As an alternative solution, the variational inference is applied to approximate p(z|x) with a tractable distribution q(z|x), so that the Kullback–Leibler (KL) divergence $D_{KL}\left[q(z|x)||p(z|x)\right]$ is minimized, which we have accomplished [24]:

(2) $$\begin{array}{ll} & D_{KL}\left[q\left(z|x\right)||p\left(z|x\right)\right]\\ & =\int q\left(z|x\right)\log\frac{q\left(z|x\right)}{p\left(z|x\right)}dz\\ & =\int q\left(z|x\right)\log q\left(z|x\right)dz-\int q\left(z|x\right)\left[\log p\left(x|z\right)+\log p\left(z\right)-\log p\left(x\right)\right]dz\\ & =\int q\left(z|x\right)\log\frac{q\left(z|x\right)}{p\left(z\right)}dz+\log p\left(x\right)-\int q\left(z|x\right)\log p\left(z|x\right)dz\\ & =D_{KL}\left[q\left(z|x\right)||p\left(z\right)\right]+\log p\left(x\right)-E_{q\left(z|x\right)}\left[p\left(z|x\right)\right] \end{array}$$

The above equation can be manipulated by maximizing the following objective loss (also known as the evidence lower bound or ELBO),

(3) $$\begin{array}{ll} loss_{vae}=\log p\left(x\right)-D_{KL}\left[q\left(z|x\right)||p\left(z|x\right)\right] & \\ =E_{q\left(z|x\right)}\left[p\left(x|z\right)\right]-D_{KL}\left[q\left(z|x\right)||p\left(z\right)\right] & \\ =loss_{vae}^{recon}+loss_{vae}^{kld} & \end{array}$$

where the left expectation term is the reconstruction loss and the right is the KL divergence (KLD) loss. Let $\tilde{x}$ be the reconstructed input; the reconstruction loss can be realized by the mean squared error $\frac{1}{N}\sum_{k}{\left\|x_{k}-{\tilde{x}}_{k}\right\|}_{2}^{2}$. The prior of the latent is usually defined as p(z)=N(0,I). Penalizing the reconstruction encourages the distribution to accurately describe the input, while penalizing the KL loss will encourage the distribution to have zero means and sufficient variances for yielding a smoothed latent space. One can see that the VAE objective induces the reconstruction and KL regularization terms from a principled Bayesian perspective. Among them, the encoder and decoder networks are built to approximate those two probability terms on the left as $p_{\theta }\left(x|z\right)$ and $q_{\phi }\left(z|x\right)$, where $q_{\phi }(z|x)=N\left(z;\mu_{\phi }(x),diag{\left[\sigma_{\phi }(x)\right]}^{2}\right)$, and θ and ϕ are the parameters of the decoder and encoder networks. respectively. In order to allow the errors to be back-propagated through the VAE network, the reparameterization trick is required; details can be found in [24].

### 2.2. Dynamic VAE

The dynamic VAE can only capture the high dimension and nonlinearity, but the underlying dynamics are lost. An alternative technique for dynamic modeling is time-wise augmentation or time lagging [25]. Instead of considering one sample $x_{k}$ at a time, time-lagged dynamic VAE works on the τ time-shifted duplicate vectors of all the variables $x_{k}(\tau )=[x_{k-\tau },x_{k-\tau +1},\ldots ,x_{k}]$, $x_{k}(\tau )\in R^{\tau \times D}$. In this way, we only need to flatten each input window-size matrix $x_{k}(\tau )$ into a vector ${\tilde{x}}_{k}(\tau )\in R^{\tau D\times 1}$, the remaining part is identical to the standard VAE:

(4) $$loss_{dvae}=E_{q_{\phi }\left(z_{k}|{\tilde{x}}_{k}(\tau )\right)}\left[p_{\theta }\left({\tilde{x}}_{k}(\tau )|z_{k}\right)\right]-D_{KL}\left[q_{\phi }\left(z_{k}|{\tilde{x}}_{k}(\tau )\right)||p_{\theta }\left(z_{k}\right)\right].$$

The dynamic VAE can model dynamics, as both the auto-correlation and the cross-correlation have been implicitly mapped into the latent space through time-wise data augmentation. The apparent advantage of this approach is its simplicity. From the view of system identification, the dynamic VAE is also analogous to dynamic PCA. One can actually judge that, if process inputs are included, the entire time-lagged VAE model can be implicitly regarded as a deep multivariate autoregressive (AR) or ARX model.

### 2.3. Recurrent VAE

The recurrent networks are designed with connections between nodes along a temporal sequence so as to deal with sequential data, and nodes can be input, hidden, or output. A traditional simple recurrent unit may suffer from exploding gradients and vanishing gradients when back-propagating errors across many time steps [26]. For this reason, two modern recurrent units, called the Long Short Term Memory (LSTM) and the Gated Recurrent Unit (GRU), will be considered in this work. Both units have internal mechanisms called gates that can regulate information flow and remember information for long time periods without having to concern themselves with the gradient problem. We first introduce the LSTM and GRU units, and then come to the LSTM-VAE and GRU-VAE.

#### 2.3.1. LSTM

First, consider LSTM: different from the dynamic VAE, the temporal sequence is here modeled with a recurrent unit. The LSTM unit is composed of a cell, an input gate, an output gate, and a forget gate. For sample k in the input sequence, the LSTM performs the following calculations at each time step [26]:

(5) $$\begin{array}{l} i_{k}=\sigma (W_{ii}x_{k}+b_{ii}+W_{hi}h_{k-1}+b_{hi}),\\ g_{k}=\tanh\left(W_{ig}x_{k}+b_{ig}+W_{hg}h_{k-1}+b_{hg}\right),\\ f_{k}=\sigma (W_{if}x_{k}+b_{if}+W_{hf}h_{k-1}+b_{hf}),\\ o_{k}=\sigma (W_{io}x_{k}+b_{io}+W_{ho}h_{k-1}+b_{ho}),\\ c_{k}=f_{k}⊙c_{k-1}+i_{k}⊙g_{k},\\ h_{k}=o_{k}⊙\tanh\left(c_{k}\right), \end{array}$$

where $g_{k}$, $c_{k}$, and $h_{k}$ are the input state (or new memory cell state), cell state (or final memory cell state), and hidden state; $i_{k}$, $f_{k}$, and $o_{k}$ are the input, forget, and output gates; σ is the sigmoid function, and ⊙ is the Hadamard product.

The gate value is used to multiply the value of the state so as to regulate the information flow for state updating. For LSTM, the input gate chooses what information is relevant to add from the current step. The forget gate inspects what is relevant to keep from the prior steps. The output gate determines what the next hidden state should be. The cell takes the previous memory state $c_{k-1}$ and performs element-wise multiplication with the forget gate. In this way, the LSTM is enabled to remember values over long time intervals.

#### 2.3.2. GRU

The GRU is a variant of LSTM [27]. For each element in the input sequence, the GRU performs the following calculations at each time step:

(6) $$\begin{array}{l} r_{k}=\sigma (W_{ir}x_{k}+b_{ir}+W_{hr}h_{k-1}+b_{hr}),\\ o_{k}=\sigma (W_{io}x_{k}+b_{io}+W_{ho}h_{k-1}+b_{ho}),\\ n_{k}=\tanh\left(W_{kn}x_{k}+b_{in}+r_{k}⊙\left(W_{hn}h_{k-1}+b_{hn}\right)\right),\\ h_{k}=\left(1-o_{k}\right)⊙n_{k}+o_{k}⊙h_{k-1}, \end{array}$$

where $r_{k}$ and $o_{k}$ are the reset and update gates, $n_{k}$ is the new memory generated, and $h_{k}$ still refers to the hidden state. GRU has used the hidden state to transfer information. It has only two gates, a reset gate and an update gate. The update gate functions quite similarly to the forget and input gates of LSTM. It actually solves the problem of how much past information should be carried forward and how much new information should be added in, whereas the reset gate is used to settle on how important the past information is for summarizing the new information memory.

### 2.4. Combining the Recurrent Unit with VAE

**Figure 1:**
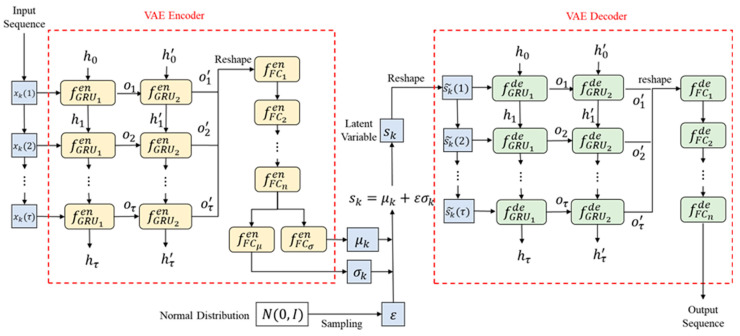
Structure of GRU-VAE.

In this part, we show how to combine the recurrent unit with VAE. For input time sequence $x_{k}(\tau )$, the LSTM can output the cell state $c_{k}$ and hidden state $h_{k}$. This work uses the cell state as the compact representation of the current system state $s_{k}$, so $s_{k}=c_{k}$, while for GRU, only the hidden state can be used, and $s_{k}=h_{k}$. In this way, the rest of the encoder layers can be readily connected to the output state $s_{k}$ of the recurrent unit so as to extract the latent $z_{k}$. The decoder has a similar structure to the encoder, except that the recurrent unit is deployed at the decoder input, which is a latent vector. In order to align with the input time sequence length, we simply use zero padding to reshape the latent vector $z_{k}$ into a sequence ${\tilde{z}}_{k}(\tau )$. Then, the state sequence flows into the recurrent unit for state sequence reconstruction, and then goes into the rest of the decoder layers for input reconstruction. Figure 1 shows the structure of GRU-VAE. Notice that the encoder has included two GRU layers, followed by n-cascaded full connection layers, and then outputs the mean and variance separately; the decoder’s architecture is inverted. The entire loss function is similar with the dynamic VAE, which is:

(7) $$loss_{rvae}=E_{q_{\phi }\left(z_{k}|x_{k}(\tau )\right)}\left[p_{\theta }\left(x_{k}(\tau )|z_{k}\right)\right]-D_{KL}\left[q_{\phi }\left(z_{k}|x_{k}(\tau )\right)||p_{\theta }\left(z_{k}\right)\right].$$

Notice that ϕ here parameterizes the entire recurrent encoder, while θ contains the parameter from the entire recurrent decoder.

## 3. Process Monitoring with VAE and Variants

In this section, we first define the fault detection methods, and then follow with the fault diagnosis mechanisms. For fault detection, three different ways will be introduced and comparatively discussed, while for fault diagnosis, the deep contribution plot and the deep reconstruction-based contribution plot will be developed.

### 3.1. Fault Detection

As a generative model, the fault detection charts can be commensurable with PCA. That is, we can monitor each sample from any of the latent, residual, and combined spaces. This part will introduce three detection implementations.

#### 3.1.1. Detection via Statistical Hypothesis

Since the normal distribution is imposed on the latent space, one can borrow the same idea as PCA by using constructed statistics when making fault detection. This is also exactly what has been done in [20]. Assume the latent feature for a new test sample $x_{t}$ ($x_{t}(\tau )$ for dynamic and recurrent VAEs) is $z_{t}$: the $T^{2}$ statistic can be derived for all VAE variants as

(8) $$T^{2}(x_{t})=z_{t}^{T}\Sigma^{-1}z_{t}$$

where Σ is the covariance matrix of the training data. The upper limit has been defined by assuming the chi-square distribution $\chi_{\alpha }{}^{2}(d)$, where d is the degree and α is the significance level. Different from the latent space, there is no definite distribution for the residual space, so here we follow [20], and only provide the $T^{2}$ index.

#### 3.1.2. Detection via Loss Density Evaluation

In traditional hypothesis test constructs, the $\chi_{\alpha }{}^{2}(d)$ statistic should rely on the validity of the latent assumption; such a mechanism can become infeasible if there exists a large deviation in real practice. Essentially, the problem lies in how to derive the upper limit, given the certain significance level. If we look back on the loss function, one can actually see that the two loss terms measure different variation aspects: the KLD term indicates the latent variation and the reconstruction manifests the residual variation. As a composite, the entire loss is a weighted function of the two, which can be viewed as the combined formulation of two spaces. In this regard, we can directly come up with a systematic fault monitoring diagram by inspecting the three loss densities. To determine the upper boundary of each loss of the training data, the kernel density estimation (KDE) can be used [28].

#### 3.1.3. Detection via Subnetwork

The above statistical test and loss inspection all require the distribution of latent/residual projections, which should depend highly on the estimation validity. To get rid of this, we introduce here a novel subnetwork detection method. The basic idea of subnetwork detection is to build two subnetworks based on latent and residual projections, which can automatically map the nominal latent or residual manifolds into the respective minimum volumes of the hyperspheres. In this way, fault detection can be readily made by comparing the distance between the sample and the hypersphere center.

First, consider the latent domain: the objective is designed to train the detection network so that the following loss can be optimized:

(9) $$loss_{ld\_vae}=\sum_{k=1}^{N}\frac{1}{\alpha \cdot N}\{\alpha \cdot R_{ld}^{2}+\max\left(0,{\left\|f_{\varsigma }^{ld}\left(z_{k}\right)-c_{ld}\right\|}_{2}^{2}-R_{ld}^{2}\right)\}$$

For the dynamic VAE, one can define a similar formulation. Since the first time sequence starts from τ, we have

(10) $$loss_{ld\_vae}=\sum_{k=\tau }^{N}\frac{1}{a\cdot (N-\tau +1)}\left\{\alpha \cdot R_{ld}^{2}+\max\left(0,{\left\|f_{\zeta }^{ld}(z_{k})-c_{ld}\right\|}_{2}^{2}-R_{ld}^{2}\right)\right\}$$

Here, α is the significance level, and $f_{\zeta }^{ld}$ denotes that subnetwork f is configured with parameter ζ. The cluster location center $c_{ld}$ and radius $R_{ld}$ are trainable parameters and can be self-tuned during the training process. One can infer that the loss optimization will learn the subnetwork, such that the majority proportion (e.g., (1−α)100%) of the latent features can be mapped around the center of the affiliated hypersphere. Once the network has been trained, the boundary has also been determined. The output of a new test sample $f_{\zeta }^{ld}(z_{t})$ can now be directly used for fault detection. The test sample is regarded as faulty in latent space if ${\left\|f_{\zeta }^{ld}(z_{t})-c_{ld}\right\|}_{2}^{2}-R_{ld}^{2}\geq 0$.

The residual detection subnetwork can be built in an analogous way. First, the residual is calculated as ${\hat{x}}_{k}=x_{k}-{\tilde{x}}_{k}$ for each sample; then, the subnetwork is built for the static VAE by optimizing the following loss:

(11) $$loss_{{}_{rd\_vae}}=\sum_{k=1}^{N}\frac{1}{a\cdot N}\left\{\alpha \cdot R_{rd}^{2}+\max\left(0,{\left\|f_{\zeta }^{rd}({\hat{x}}_{t})-c_{rd}\right\|}_{2}^{2}-R_{rd}^{2}\right)\right\}.$$

For dynamic and recurrent VAEs, we also have:

(12) $$loss_{{}_{rd\_dvae}}=\sum_{k=\tau }^{N}\frac{1}{a\cdot (N-\tau +1)}\left\{\alpha \cdot R_{rd}^{2}+\max\left(0,{\left\|f_{\zeta }^{rd}({\hat{x}}_{t})-c_{rd}\right\|}_{2}^{2}-R_{rd}^{2}\right)\right\}.$$

Note that, for recurrent VAEs, one needs to first flatten the window-size residual matrix into a vector by concatenating each time channel and then feeding the entire vector into the subnetwork. After that, a test sample can be regarded as a fault in residual space if ${\left\|f_{\zeta }^{rd}({\hat{x}}_{t})-c_{rd}\right\|}_{2}^{2}-R_{rd}^{2}\geq 0$.

#### 3.1.4. Remarks on Three Fault Detection Methods

**Figure 2:**
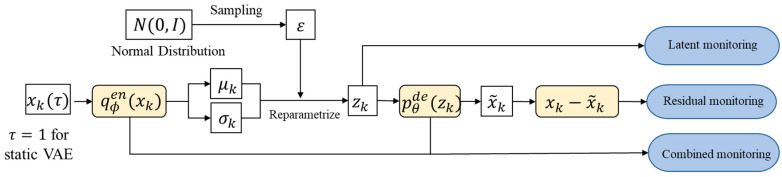
Fault detection flowchart.

Essentially, all three detection methods share the same logic, and the fault has been investigated from the latent and residual domains. A general fault detection flowchart has been given in Figure 2. Technically, the traditional statistic method and the loss inspection method are density-based methods. The advantage of density-based methods is that their performance can be guaranteed. In contrast, the subnetwork method is formed within the distance-based framework. By using the neural network as the detection module, the detector can be trained effectively on large data with the stochastic gradient descent optimizer, and can also be deployed flexibly on the VAE backbone for straightforward fault detection, without resorting to density estimations. Additionally, the threshold can be well determined once the network training is completed. However, one potential issue with this method is that the detection performance can become inferior if the network is improperly designed or badly trained. Through this remark, we hope to probe the pros and cons for these fault detection methods, so as to provide an overall qualitative assessment before practicing the fault detection.

### 3.2. Fault Diagnosis

After the detection of a fault, diagnosis further estimates the fault size and location. Generally speaking, fault detection undertakes the forward-flow information in the network while diagnosis turns to the backward-flow evaluation. In this section, the contribution plot method is first developed for fault localization. Then, the reconstruction-based contribution is further proposed.

#### 3.2.1. Deep Contribution Plot

As aforementioned, the deep networks are essentially deep functions assembled with parameterized units under the differentiable programming paradigm. During the model training, the optimizer aims at searching the hilly landscape for parameter space, as the negative gradient points to the error descent direction in each iteration. By this reasoning, the contribution plots for the test sample $x_{t}$ can be easily derived for the static VAE as:

(13) $$\begin{array}{l} \mathrm{Latent}\;\mathrm{domain}:\;dCP_{ld}(x_{t}^{i})=\frac{\partial loss_{vae}^{kld}(x_{t})}{\partial x_{t}^{i}},\\ \mathrm{Residual}\;\mathrm{domain}:\;dCP_{rd}(x_{t}^{i})=\frac{\partial loss_{vae}^{recon}(x_{t})}{\partial x_{t}^{i}},\\ \mathrm{Combined}\;\mathrm{domain}:dCP_{com}(x_{t}^{i})=\frac{\partial loss_{vae}(x_{t})}{\partial x_{t}^{i}}, \end{array}$$

where $x_{t}^{i}$ is the ith variable of $x_{t}$, $dCP_{ld}(x_{t}^{i})$, $dCP_{rd}(x_{t}^{i})$, and $dCP_{com}x(x_{t}^{i})$ are the deep contribution plots for the latent, residual, and combined space domains; each indicator reports the potentially increased error with respect to the individual loss domain. One can infer that the derivatives are easily obtained by the chain rule of gradients with back-propagation in the multiplayer network. For dynamic and recurrent VAEs, one only has to replace the input $x_{t}$ with a test sequence $x_{t}(\tau )$, and the definitions of the contribution maps remain the same; we omit them here for simplicity.

To have a further understanding, some discussions are made to compare with contribution plots of PCA. First, we briefly revisit PCA. PCA seeks the principal and residual subspace projections by performing the eigen-decomposition of the covariance matrix S as

(14) $$S=[\begin{array}{cc} P & \tilde{P} \end{array}]\left[\begin{array}{cc} \Lambda & 0\\ 0 & \tilde{\Lambda } \end{array}\right][\begin{array}{cc} P & \tilde{P} \end{array}]$$

Then, the latent subspace $\hat{x}$ and residual subspace $\tilde{x}$ can be projected with P and $\tilde{P}$ as

(15) $$\{\begin{array}{c} {\hat{x}}_{t}=PP^{T}x_{t}=Cx_{t}\\ {\tilde{x}}_{t}=\tilde{P}{\tilde{P}}^{T}x_{t}=\tilde{C}x_{t} \end{array},$$

The monitoring index for PCA is generally defined as

(16) $$\mathrm{Index}(x_{t})=x_{t}^{T}Mx_{t},$$

where *M* is different for $T^{2}$, *SPE*, and the combined index φ [4,28,29]:

(17) $$M=\{\begin{array}{c} P\Lambda^{-1}P^{T}\\ \tilde{C}\\ \tilde{C}/\delta^{2}+D/\tau^{2} \end{array}\begin{array}{c} for\;T^{2}\\ for\;SPE\\ for\;\varphi \end{array}$$

Accordingly, the contribution plot can be derived for each index by simply taking the first derivative of (3), with respect to each variable, as $CP(x_{t})=\frac{\partial \mathrm{Index}(x_{t})}{\partial x_{i}}$. In this view, the quadratic monitoring index acts like the loss function in (13), where the contribution reveals the potentially increased loss in each of the three domains. Comparing this with the above VAE definitions, one may also speculate that the contribution plots of the VAE and PCA are closely connected, and the VAE plots take the PCA as a special case.

#### 3.2.2. Deep Reconstruction-Based Contribution

**Figure 3:**
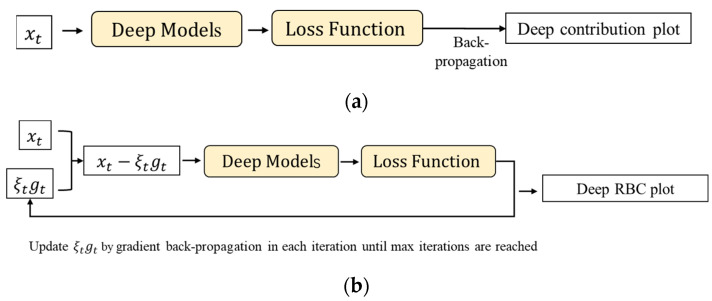
Flowcharts for (**a**) the deep contribution plot and (**b**) the deep reconstruction-based contribution plot.

The deep contribution plots are derived based on the one-step derivative of loss functions, which may not always ensure the correctness of a diagnosis (see Figure 3). This is due to two reasons. Firstly, the parameter optimization space can have multiple peaks, and the one-step derivative may only point to a local extremism. Second, the deep model is merely an approximation function of a real system, and hence the raw gradient is also noisy for the contribution plot visualization.

To clearly reveal the responsible features, the deep reconstruction-based contribution (dRBC) approach is now proposed; the basic idea is also inspired by PCA [4]. Assume a fault has happened in the ith sensor of sample $x_{t}$: let ξ be the fault direction and g be the fault magnitude; the entries assigned with ones in the direction vector indicate faulty variable items and zeros imply normal variables. The reconstructed vector along the direction is ${\hat{x}}_{t}=x_{t}-\xi_{t}g_{t}$. The task of reconstruction is to find $\xi_{t}g_{t}$ so that the fault detection index is minimized.

In theory, the fault detection index can be any above-mentioned fault detection unit. For this work, we recommend that the dRBC mechanism could be more effective under the subnetwork detection strategy through end-to-end loss gradient propagation. The reasons are comprehensible: On the one hand, the loss functions are the criterion for model training. In other words, they are empirical indicators for judging the status of a complex process. On the other hand, the backward inference by the gradients can be performed on the entire network model with layer-by-layer inference using the chain rule. Therefore, it is more desirable to use the loss function, rather than statistics, to make the diagnostic improvement and interpretation.

Technically, the dRBC objective is to push the index function toward the threshold. Therefore, the general dRBC procedure for static VAEs can be formulated as to continuously optimize the objective

(18) $$\min\;\mathrm{Index}(x_{t}-\xi_{t}g_{t}),$$

until $\mathrm{Index}(x_{t}-\xi_{t}g_{t})\leq Threshold$ is satisfied. Here, the ith element in $g_{t}$ is $g_{t}^{i}=\frac{\partial \mathrm{Index}(x_{t})}{\partial x_{i}}$.

For dynamic VAEs, the objective is

(19) $$\min\;\mathrm{Index}({\tilde{x}}_{t}(\tau )-{\tilde{\xi }}_{t}g_{t})$$

For recurrent VAEs, the objective can be written as

(20) $$\min\;\mathrm{Index}(x_{t}(\tau )-\Xi_{t}G_{t})$$

where $\Xi_{t}$ is a window-sized fault direction matrix at time t with length τ, and $G_{t}$ is the corresponding fault magnitude matrix. From the definition, one can judge that the deep RBC can be regarded as the enhancement of the deep CP counterpart with multiple optimizations.

#### 3.2.3. The Deep RBC Implementation

Based on the above theory, this part presents the implementation details. During the experiment, we have found that the RBC may usually take hundreds or thousands of iterations to hit the convergence in practice. Hence, the main concern turns to the real-time application of the deep RBC diagram. One can consider the iterative optimization for each variable at a time, but the efficiency will be extremely low. As an alternative, a novel network input retraining-based strategy is implemented that can optimize the whole variable set. Specifically, let $\delta_{t}$ be the negative reconstruction term, which can be defined as

(21) $$\delta_{t}=\{\begin{array}{c} -\xi_{t}g_{t},\mathrm{for}\;\mathrm{static}\;\mathrm{VAE}\\ -{\tilde{\xi }}_{t}g_{t},\mathrm{for}\;\mathrm{dynamic}\;\mathrm{VAE}\\ -\Xi_{t}g_{t},\mathrm{for}\;\mathrm{recurrent}\;\mathrm{VAE} \end{array}$$

The dRBC attempts to optimize δ so as to minimize the loss index, so we will modify the VAE network input in two parts: the original input $x_{t}$ (or $x_{t}(\tau )$ for dynamic and recurrent VAEs) plus the negative reconstruction term δ. Accordingly, only δ is trainable, while the rest of the network is still fixed as untrainable. Through this setting, the network retraining procedure can be launched under the predefined optimizer and loss functions, with the goal of searching for the input perturbation that can reduce the total model loss of the reconstructed data.

Different than [4], where only a one-step derivative is required for the linear projection PCA model, multiple derivative and updating steps should be engaged for the above loss minimization, as our deep network is a highly nonlinear projection model. Please notice that sometimes the fault may have large magnitudes, and the above optimization may not always converge below the threshold within the affordable time. In this case, one may set the maximum iteration number and obtain a trade-off solution for diagnosis.

The network input retraining strategy uses the entire deep function to estimate the unexpected deviations from the normal status. Although the retraining procedure usually can be quite fast, it is still noteworthy to introduce two tricks to make the further acceleration. The first trick is to utilize the estimated deviation $\delta_{t-1}$ from the last time as the potential initialization for the current optimization. This is feasible, as most faults have temporal accumulation effects. The second trick is to perform multithreaded programming, with each thread bearing one retraining process. By assuming that the internal sampling is $T_{s}$ and the worst estimation time elapsed for δ is $T_{\delta }$, where $T_{s}<T_{\delta }$, the required program thread number $N_{thread}$ can be estimated as

(22) $$N_{thread}=\left\lceil \frac{T_{\delta }-T_{s}}{Ts}\right\rceil$$

where ⌈.⌉ is the ceiling function. In this way, one can ensure the immediate availability of at least one empty thread for each new test sample, and the time lag for the diagnosis can be fixed at $T_{\delta }-T_{s}$ for all samples without any time lagging accumulations.

## 4. Case Study

As a typical complex industrial process with nonlinear and dynamic characteristics, the TE process was extracted from a real chemical plant and has been widely used for fault simulation and process monitoring demonstrations [29,30]. In this section, the VAE and its variants will be comparatively studied on the revised TE process [31].

### 4.1. Data and Model

The original process has 12 manipulated variables and 41 measurement variables. In this study, we neglect those constant or quality variables, and a total of 31 variables have been used as [32]. For model development, 10,000 samples are gathered under the normal operation. For model validation, the explicit fault descriptions for all engaged 28 fault cases can be found in [31]. Each fault is collected as a 1000-length data sequence and the fault signals have been introduced after the 300th sampling time.

As for the deep model specifications, the architecture details for VAEs, dynamic VAEs, and recurrent VAEs are given in Table 1, all models in this work are technically implemented with python, and the library for deep learning is Pytorch. The abbreviations in the table follow the definitions as:

- RNN(*d*) is the RNN unit (GRU or LSTM) with hidden dimension *d*;
- FC(m) is the full connection with m outputs;
- Flatten is the reshaping of the matrix into a vector;
- Padding is the zero padding operation.

### 4.2. Study on Fault Detection

In this part, the three introduced fault detection diagrams will be comparatively studied. To set the upper control limits, tolerance rate α of false alarms is universally set at the level of 0.03. The fault detection results for PCA, AE, VAE, dynamic VAE, GRU-VAE, and LSTM-VAE have been listed in Table 2, Table 3, Table 4, Table 5 and Table 6, the false alarm rate (FAR) and (fault detection rate) are used as performance monitoring indicators.

**Figure 4:**
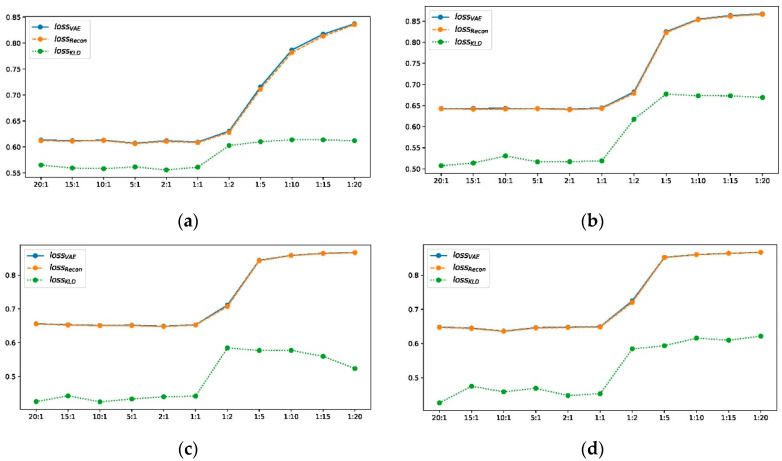
Detection accuracy with different VAE loss weight ratios.

One can draw several major conclusions from the detection tables. First of all, from the general view of the model architectures, the deep generative models of the AE and the VAE greatly outperform the shallow model of PCA. This result reveals the competitive advantage of deep models for complex process modeling and representation, which, in turn, brings great benefits to the monitoring venture. Second, given the same VAE archetype, one can easily judge that the dynamic and recurrent deep models triumph over the static counterpart. Both dynamic and recurrent VAEs impose the reasoning of spatial and temporal domains to improve the fault detection abilities. In addition, LSTM-VAE and GRU-VAE generally achieve similar detection results. Notice that although here we only display the results by setting the weight ratio to 1:20 for the latent KL loss and reconstruction loss in Equation (3), the same conclusion can be derived by varying different weight ratios, as shown in Figure 4. The blue, orange, and green lines represent the detection rates from different loss spaces under various weight ratios of KL/Reconstruction loss. One can see that the overall detection rates of the residual and combined spaces are basically at the same level, and both should outperform the latent space counterpart. Finally, if one considers comparing those three monitoring methods for the VAE, one can find that the $T^{2}$ with a poor effect can only monitor the latent variations, which is not amenable and sufficient in most fault cases. Alternatively, the loss density estimation method and detection subnetwork method leverage both latent and residual spaces and will have more desirable results. Separately, the latent detection subnetworks may not have the same detection rates as the KLD loss density. However, the residual subnetworks notably show comparable fault detection rates, as reported in dynamic and recurrent VAEs. Therefore, our results demonstrate that both density-based and distance-based methods are favorable for fault detection with deep VAE models.

### 4.3. Study on Fault Diagnosis

After the fault detection, this part will make the comparative study of fault diagnosis using the designed deep contribution plot and deep reconstruction-based contribution. To comprehensively make the investigation, we will successively evaluate three impacting factors: the models, the loss weights, and the epochs for deep reconstruction.

**Figure 5:**
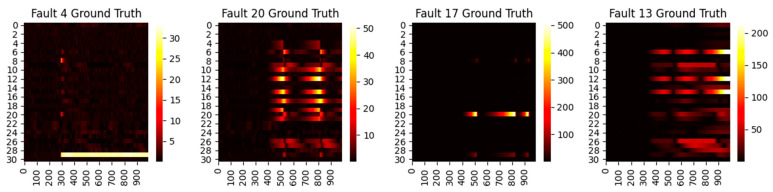
Ground truth heat maps of faults 4, 20, 17, and 13. The horizontal axis denotes the sampling time, while the vertical axis indicates the variable number.

**Figure 6:**
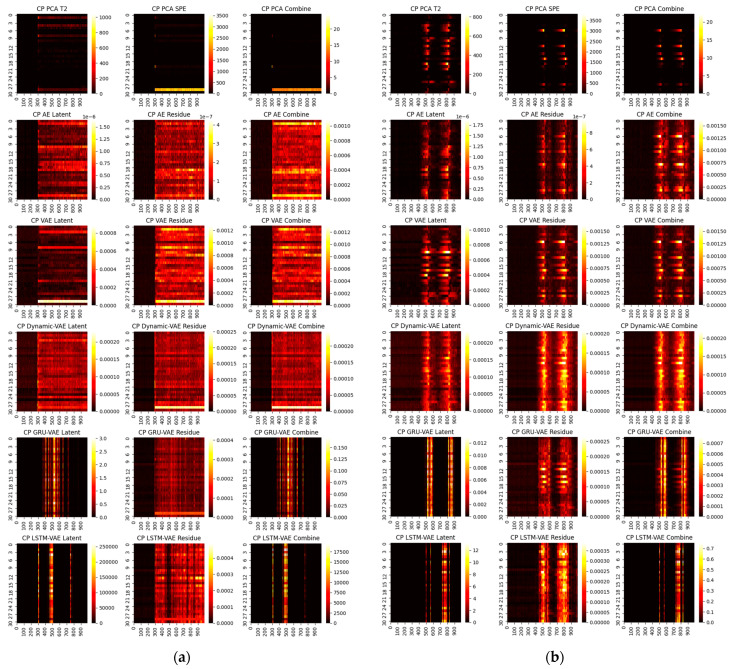
CP contribution plots for small faults: (**a**) fault 4, (**b**) fault 20.

**Figure 7:**
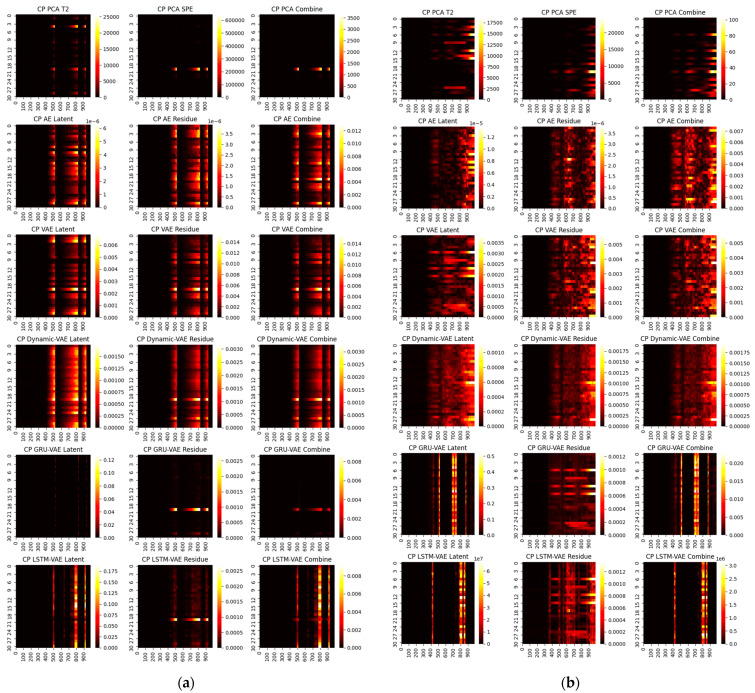
CP contribution plots for large faults: (**a**) fault 17, (**b**) fault 13.

**Figure 8:**
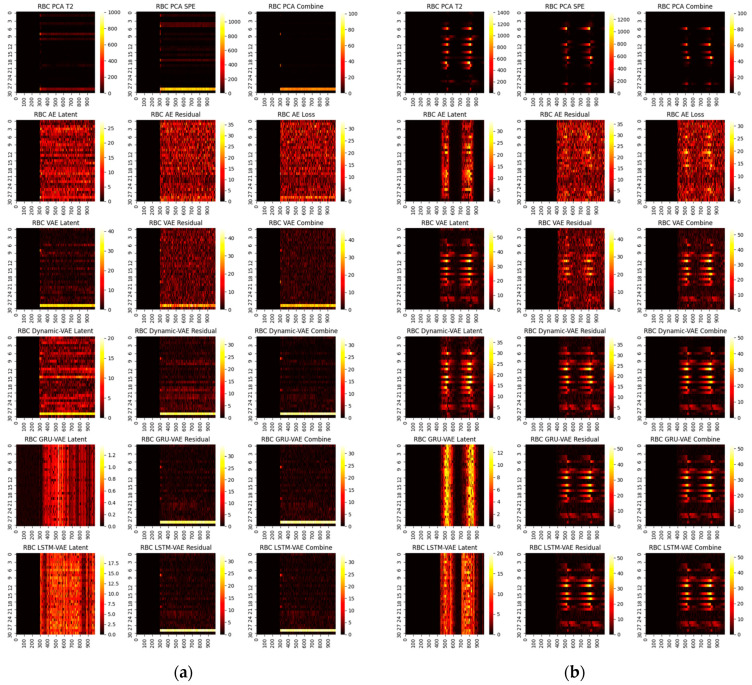
RBC contribution plots for small faults: (**a**) fault 4, (**b**) fault 20.

**Figure 9:**
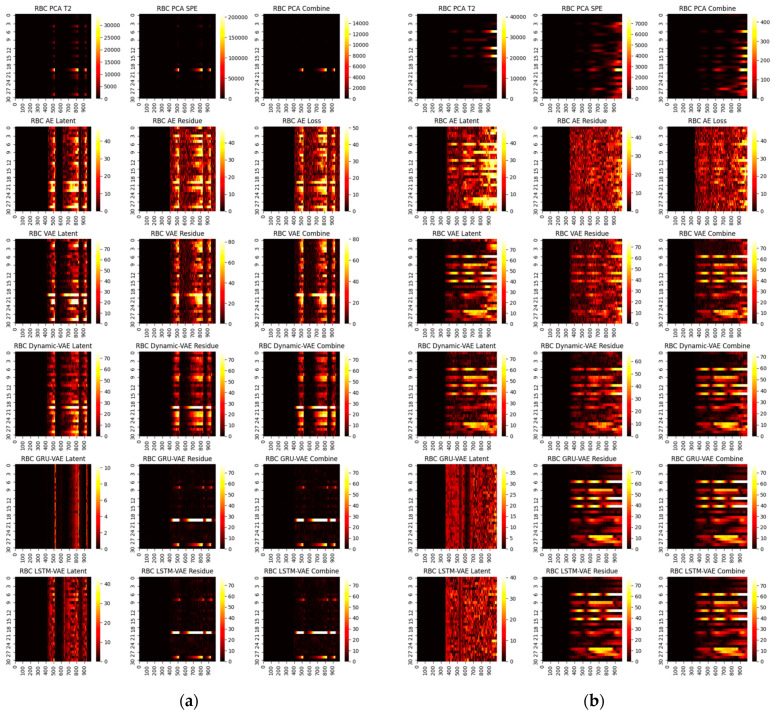
RBC contribution plots for large faults: (**a**) fault 17, (**b**) fault 13.

First, we consider the diagnosis results with different models. A total of four representative faults have been selected, and the ground truth heat maps are shown in Figure 5. As can be seen, faults 4 and 20 have relatively small magnitudes, while faults 17 and 13 are faults with large magnitudes. In addition, faults 4 and 17 only happen in a single variable, whereas 20 and 13 are multiple faults. To make the fair comparison among deep models, the loss weights are fixed at 1:20 and the epochs in the deep reconstruction are all set at 3000. With this setting, the derived contribution plots and RBC plots are shown in Figure 6, Figure 7, Figure 8 and Figure 9.

**Figure 10:**
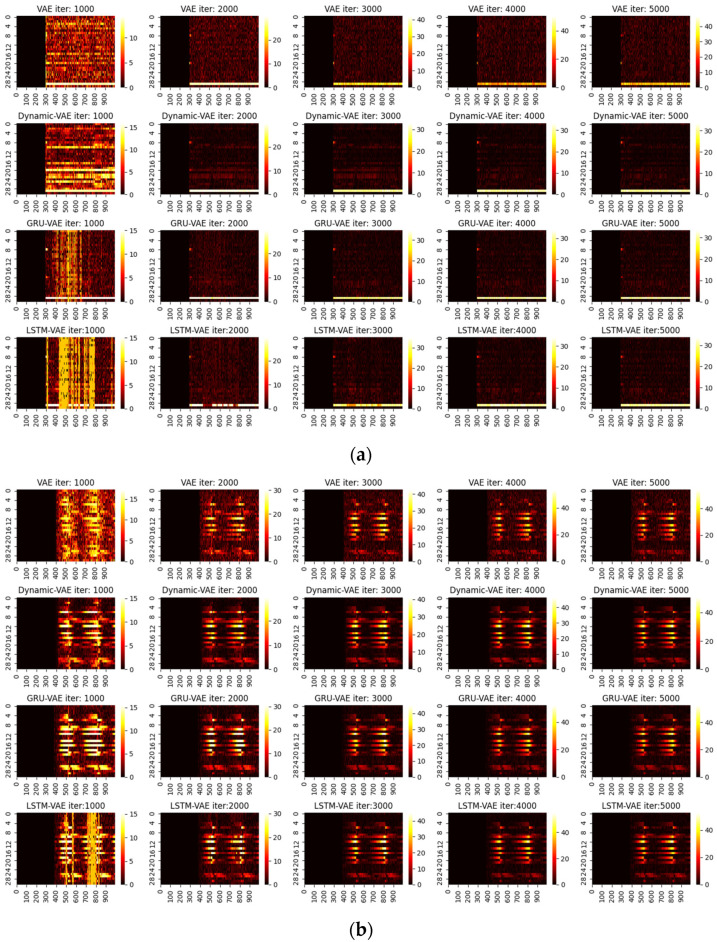

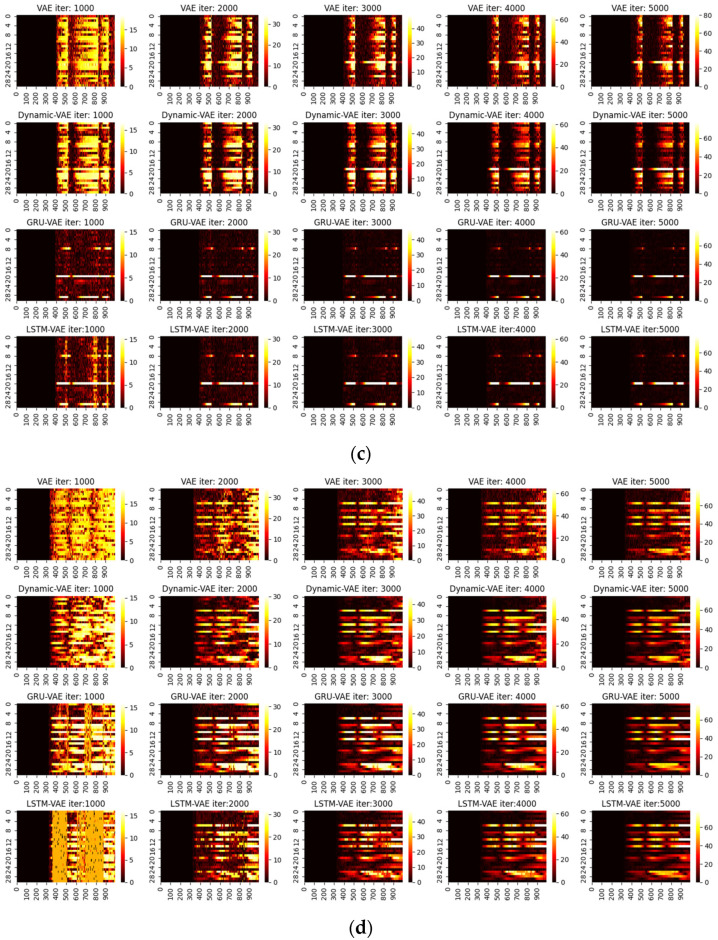
RBC contribution plot of VAE, Dynamic-VAE, GRU-VAE, and LSTM-VAE, with respect to max epochs of 1000, 2000, 3000, 4000, and 5000: (**a**) Fault 4, (**b**) Fault 20, (**c**) Fault 17, (**d**) Fault 13.

One can infer from the CP plots (Figure 6 and Figure 7) that several diagnosis plots actually show large deviations from the fundamental truth. The PCA can usually obtain a meaningful diagnosis in the residual and combined domains, but may, more or less, have deviations against the truth plots in most faults due to the smearing effects. The deep models have a similar issue, and one can speculate that the noisy gradients may even severely overwhelm those informative gradient flows from the responsible fault nodes during the back-propagation. Fortunately, as can be seen, such a noisy gradient problem in deep models can be largely alleviated by using the devised RBC scheme. The deep reconstruction is an enhanced implementation for contribution analysis with iterative optimizations. Specifically, one can judge from Figure 8 and Figure 9 that significant improvements are found in all domains. Typically, the fault magnitude can be well determined once the detection indicator has been pulled close to the threshold. Please note that the estimation speed and accuracy are highly associated with both the model and the underlying fault magnitude. To make the investigation, we use the combined domain as the example, and the RBC contribution plots over various optimization epochs have been given in Figure 10.

**Figure 11:**
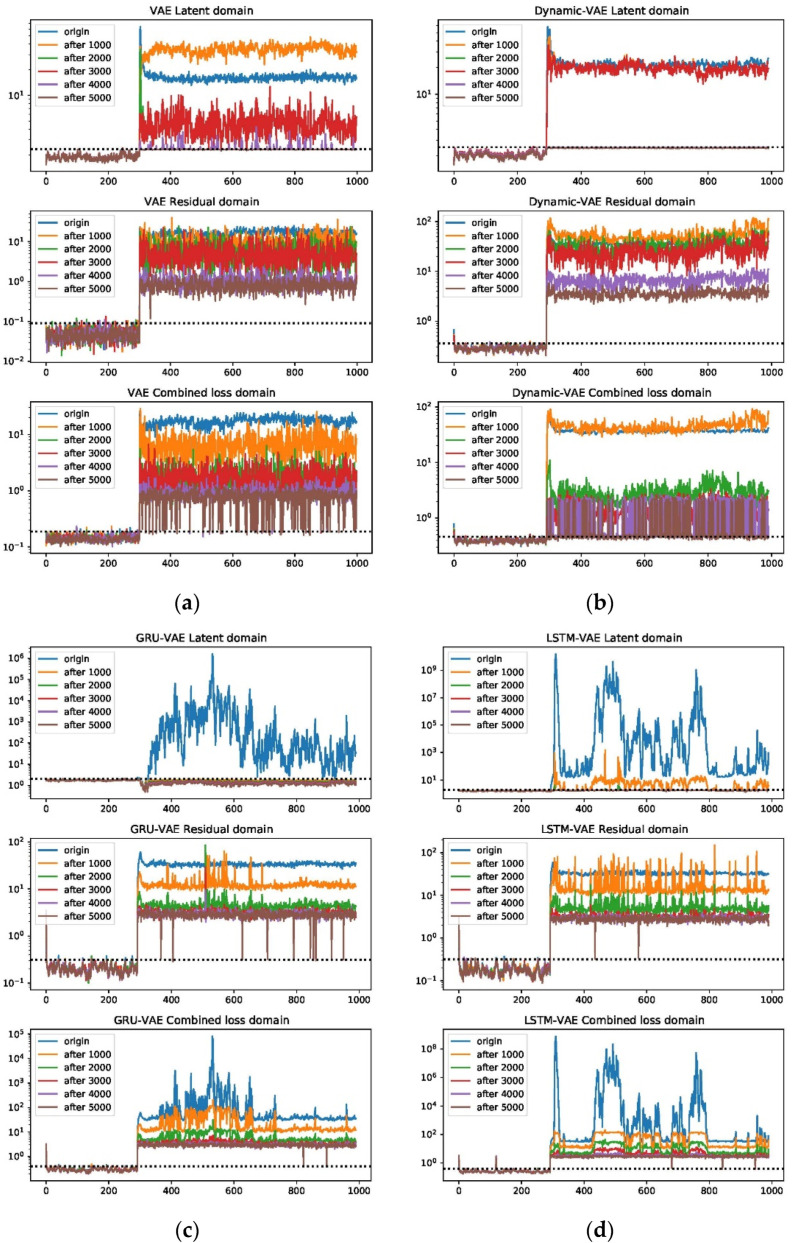
Combined, latent, and residue domain loss plots of VAE (**a**), Dynamic-VAE (**b**), GRU-VAE (**c**), and LSTM-VAE (**d**), with respect to 1000, 2000, 3000, 4000, and 5000 epochs on fault 20.

One can see that the RBC plots from the static VAE can be very noisy even after deep optimizations. By contrast, VAE variants with spatial-temporal compositions can perform much better; this can be especially verified for GRU-VAE. Apart from that, one should note that the estimated fault magnitude can match the fault well in around 3000 steps for small faults such as 4 and 20. Using fault 20 as an example, the entire loss tendency after reconstruction under different optimization iterations has been shown in Figure 11. One can judge that the latent domain can be successfully recovered to the normal status. However, this is not the case for the residual and combined domains. Both domains can give rise to strong and accurate results in fault detection, but the loss index can be hardly regulated into the normal zone. This is particularly significant in large fault cases.

For large faults, even 5000 steps can only lead to an approximated estimation. This should be originated from the fact that the gradient values are very small during each iteration, which, in turn, may lead the optimizer to get stuck easily into the local maximum when disentangling the large fault. Nevertheless, compared with PCA and deep CP, which may yield inappropriate conclusions, the deep RBC can achieve results with more refined and desirable diagnosis charts. Ultimately, we can conclude here that the deep RBC charts deployed under the GRU-VAE/LSTM-VAE archetype are very appealing and promising for discerning abnormal events in large and complex industrial processes.

## 5. Conclusions

Deep networks are believed to hold great potential to resolve early fault detection and accurate diagnosis. To attain that, this work focuses on the comprehensive study of VAE and its variants (with LSTM and GRU compositions) on process monitoring. We first establish three detection strategies, including statistics, loss density investigations, and the subnetwork methods, for different monitoring domains. Then, the deep contribution plot and reconstruction-based contribution plot have been proposed for fault diagnosis. Finally, the deep modeling and monitoring techniques are comparatively evaluated on the industrial TE benchmark. Through this work, we not only define a systematic monitoring paradigm, but also help promote the understanding of deep VAE models in solving pressing safety problems of complex processes.

While the main advantages of the deep learning-based monitoring method can be easily seen from this work, there are several outlooks. As the future work, more efforts will be made from two folds. On the one hand, diagnosis performance should be further modified for large faults so as to enhance the deep model interpretability. On the other hand, quantitative analysis is also required for the detectability and diagnosability analysis [32,33] of various deep models. In this way, we hope that deep models can make a better service on the monitoring of complex industrial process systems.

## Figures and Tables

**Table 1:** Deep architectures for all VAE models.

	VAE	Dynamic VAE	GRU-VAE	LSTM-VAE
Input	$x_{k}$	$x_{k}(\tau )$	$x_{k}(\tau )$	$x_{k}(\tau )$
Preprocess	--	Flatten	--	--
Encoder	FC (400)	FC (800)	GRU (200)	LSTM (200)
	FC (400)	FC (800)	GRU (200)	LSTM (200)
	FC (20), FC (20)	FC (20), FC (20)	FC (800)	FC (800)
			FC (20), FC (20)	FC (20), FC (20)
Decoder	FC (400)	FC (800)	Padding	Padding
	FC (400)	FC (800)	GRU (200)	LSTM (200)
			GRU (200)	LSTM (200)
			FC (800)	FC (800)

**Table 2:** Fault detection by PCA and AE.

ID	PCA			AE		
	T^2^	SPE	Com	loss_AE_	subnet_l_	subnet_r_
1	0.989	0.993	0.993	0.994	0.984	0.994
2	0.943	0.951	0.960	0.990	0.911	0.983
3	0.033	0.094	0.151	0.913	0.011	0.843
4	0.999	0.999	0.999	0.997	0.997	0.997
5	0.026	0.063	0.091	0.919	0.009	0.847
6	0.999	0.999	0.999	0.997	0.997	0.997
7	0.999	0.999	0.999	0.997	0.997	0.997
8	0.883	0.897	0.904	0.921	0.870	0.917
9	0.097	0.111	0.234	0.859	0.060	0.807
10	0.729	0.881	0.890	0.921	0.583	0.921
11	0.957	0.979	0.987	0.990	0.910	0.990
12	0.471	0.540	0.743	0.961	0.313	0.954
13	0.939	0.954	0.953	0.960	0.937	0.959
14	0.967	0.989	0.989	0.990	0.914	0.989
15	0.014	0.027	0.043	0.923	0.013	0.854
16	0.053	0.043	0.126	0.003	0.033	0.001
17	0.791	0.854	0.854	0.861	0.757	0.859
18	0.493	0.600	0.666	0.699	0.446	0.691
19	0.926	0.963	0.967	0.986	0.897	0.984
20	0.830	0.857	0.857	0.890	0.816	0.881
21	0.059	0.043	0.124	0.004	0.034	0.004
22	0.079	0.136	0.236	0.827	0.034	0.781
23	0.059	0.061	0.179	0.506	0.043	0.389
24	0.847	0.783	0.893	0.914	0.724	0.911
25	0.527	0.901	0.940	0.974	0.166	0.971
26	0.813	0.901	0.911	0.954	0.680	0.949
27	0.653	0.919	0.930	0.963	0.644	0.960
28	0.054	0.029	0.086	0.869	0.023	0.823
Average	0.579	0.627	0.668	0.849	0.529	0.831
FAR	0.023	0.017	0.030	0.010	0.007	0.010

**Table 3:** Fault detection by VAE.

ID	Detection Index					
	T^2^	loss_kld_	loss_recon_	loss_VAE_	subnet_l_	subnet_r_
1	0.990	0.990	0.994	0.994	0.986	0.993
2	0.940	0.946	0.984	0.986	0.914	0.983
3	0.039	0.027	0.873	0.873	0.023	0.697
4	0.997	0.997	0.997	0.997	0.997	0.997
5	0.017	0.024	0.853	0.841	0.019	0.704
6	0.997	0.997	0.997	0.997	0.997	0.997
7	0.997	0.997	0.997	0.997	0.997	0.997
8	0.879	0.891	0.914	0.913	0.881	0.913
9	0.077	0.109	0.791	0.800	0.044	0.671
10	0.743	0.814	0.917	0.917	0.710	0.919
11	0.950	0.964	0.990	0.990	0.936	0.990
12	0.539	0.576	0.953	0.951	0.279	0.926
13	0.937	0.943	0.959	0.957	0.939	0.954
14	0.984	0.987	0.990	0.990	0.957	0.989
15	0.001	0.009	0.837	0.813	0.011	0.671
16	0.049	0.041	0.010	0.023	0.023	0.019
17	0.823	0.846	0.859	0.859	0.779	0.859
18	0.544	0.550	0.691	0.693	0.444	0.680
19	0.919	0.933	0.984	0.986	0.901	0.981
20	0.829	0.834	0.881	0.879	0.816	0.886
21	0.049	0.043	0.010	0.020	0.020	0.019
22	0.036	0.071	0.783	0.801	0.044	0.684
23	0.050	0.063	0.396	0.440	0.034	0.294
24	0.819	0.841	0.914	0.913	0.589	0.907
25	0.597	0.731	0.973	0.974	0.396	0.971
26	0.790	0.876	0.946	0.946	0.666	0.943
27	0.760	0.861	0.961	0.963	0.641	0.957
28	0.031	0.039	0.823	0.816	0.031	0.693
Average	0.585	0.607	0.831	0.833	0.538	0.796
FAR	0.007	0.010	0.013	0.012	0.009	0.020

**Table 4:** Fault detection by Dynamic VAE.

ID	Detection Index					
	T^2^	loss_kld_	loss_recon_	loss_VAE_	subnet_l_	subnet_r_
1	0.990	0.990	0.993	0.993	0.990	0.993
2	0.947	0.954	0.986	0.986	0.949	0.986
3	0.043	0.137	0.984	0.986	0.029	0.966
4	0.997	0.997	0.997	0.997	0.997	0.997
5	0.037	0.103	0.996	0.996	0.039	0.997
6	0.996	0.997	0.997	0.997	0.996	0.997
7	0.997	0.997	0.997	0.997	0.997	0.997
8	0.894	0.896	0.927	0.926	0.896	0.921
9	0.153	0.254	0.891	0.893	0.091	0.881
10	0.850	0.883	0.921	0.921	0.840	0.920
11	0.974	0.984	0.987	0.987	0.980	0.987
12	0.766	0.840	0.967	0.967	0.664	0.967
13	0.940	0.946	0.957	0.957	0.943	0.953
14	0.983	0.984	0.987	0.987	0.981	0.989
15	0.017	0.054	0.996	0.996	0.021	0.996
16	0.077	0.046	0.011	0.010	0.024	0.016
17	0.841	0.844	0.857	0.857	0.839	0.853
18	0.619	0.656	0.710	0.710	0.593	0.710
19	0.956	0.966	0.984	0.984	0.957	0.983
20	0.840	0.844	0.884	0.883	0.839	0.881
21	0.076	0.044	0.007	0.011	0.021	0.021
22	0.126	0.256	0.870	0.871	0.057	0.861
23	0.114	0.124	0.566	0.583	0.043	0.506
24	0.867	0.887	0.910	0.911	0.801	0.907
25	0.819	0.894	0.970	0.970	0.721	0.970
26	0.897	0.914	0.957	0.957	0.874	0.954
27	0.869	0.946	0.963	0.963	0.793	0.960
28	0.070	0.156	0.924	0.924	0.059	0.926
Average	0.634	0.664	0.864	0.865	0.608	0.861
FAR	0.009	0.003	0.021	0.017	0.034	0.018

**Table 5:** Fault detection by LSTM-VAE.

ID	Detection Index					
	T^2^	loss_kld_	loss_recon_	loss_VAE_	subnet_l_	subnet_r_
1	0.987	0.986	0.991	0.991	0.974	0.993
2	0.931	0.941	0.981	0.983	0.913	0.986
3	0.019	0.097	0.977	0.977	0.010	0.981
4	0.996	0.996	0.997	0.997	0.996	0.997
5	0.011	0.101	0.996	0.996	0.010	0.997
6	0.994	0.993	0.997	0.997	0.991	0.997
7	0.996	0.997	0.997	0.997	0.994	0.997
8	0.854	0.857	0.923	0.923	0.844	0.924
9	0.097	0.104	0.883	0.883	0.036	0.887
10	0.829	0.840	0.921	0.921	0.790	0.923
11	0.964	0.984	0.987	0.987	0.924	0.990
12	0.560	0.587	0.966	0.966	0.346	0.966
13	0.934	0.937	0.957	0.957	0.936	0.960
14	0.229	0.963	0.987	0.987	0.097	0.987
15	0.007	0.046	0.996	0.996	0.009	0.996
16	0.040	0.031	0.016	0.016	0.019	0.011
17	0.831	0.830	0.854	0.854	0.829	0.860
18	0.584	0.624	0.691	0.691	0.506	0.699
19	0.910	0.939	0.983	0.983	0.881	0.987
20	0.823	0.834	0.879	0.879	0.816	0.880
21	0.047	0.036	0.021	0.023	0.019	0.031
22	0.034	0.120	0.873	0.873	0.014	0.879
23	0.060	0.084	0.636	0.641	0.019	0.761
24	0.821	0.847	0.910	0.910	0.667	0.913
25	0.544	0.823	0.971	0.971	0.376	0.976
26	0.877	0.894	0.954	0.954	0.797	0.960
27	0.761	0.921	0.960	0.960	0.666	0.961
28	0.024	0.066	0.923	0.924	0.006	0.930
Average	0.563	0.624	0.865	0.866	0.517	0.872
FAR	0.005	0.000	0.024	0.021	0.009	0.017

**Table 6:** Fault detection by GRU-VAE.

ID	Detection Index					
	T^2^	loss_kld_	loss_recon_	loss_VAE_	subnet_l_	subnet_r_
1	0.977	0.976	0.990	0.991	0.973	0.993
2	0.937	0.923	0.986	0.986	0.934	0.986
3	0.019	0.107	0.977	0.979	0.024	0.983
4	0.976	0.956	0.997	0.997	0.979	0.997
5	0.013	0.079	0.996	0.996	0.040	0.996
6	0.997	0.981	0.997	0.997	0.994	0.997
7	0.997	0.997	0.997	0.997	0.999	0.997
8	0.870	0.880	0.926	0.926	0.873	0.930
9	0.110	0.171	0.881	0.881	0.086	0.887
10	0.866	0.893	0.924	0.924	0.873	0.929
11	0.779	0.589	0.987	0.987	0.789	0.989
12	0.653	0.723	0.966	0.966	0.584	0.969
13	0.933	0.957	0.954	0.956	0.943	0.956
14	0.723	0.439	0.987	0.987	0.761	0.987
15	0.016	0.050	0.996	0.996	0.021	0.996
16	0.064	0.047	0.007	0.007	0.046	0.024
17	0.839	0.640	0.854	0.854	0.817	0.860
18	0.543	0.604	0.697	0.699	0.553	0.710
19	0.944	0.961	0.983	0.983	0.943	0.986
20	0.836	0.844	0.880	0.880	0.836	0.883
21	0.051	0.046	0.016	0.017	0.044	0.036
22	0.073	0.224	0.870	0.870	0.056	0.874
23	0.069	0.079	0.680	0.686	0.060	0.779
24	0.809	0.764	0.910	0.910	0.766	0.911
25	0.664	0.601	0.970	0.970	0.601	0.969
26	0.889	0.906	0.956	0.957	0.881	0.957
27	0.364	0.327	0.960	0.960	0.374	0.964
28	0.023	0.116	0.924	0.924	0.043	0.929
Average	0.573	0.567	0.867	0.867	0.568	0.874
FAR	0.008	0.014	0.030	0.028	0.016	0.028

## Data Availability

Not applicable.

## Abbreviations

Abbreviations in alphabetical order

AE	Autoencoder
AR	Autoregressive
CNN	Convolutional Neural Network
DNN	Deep Neural Network
dCP	deep Contribution Plot
dRBC	deep Reconstruction-Based contribution
GRU	Gated Recurrent Unit
KDE	Kernel Density Estimation
KLD	Kullback Leibler Divergence
LSTM	Long Short Time Memory
MWPCA	Moving Window PCA
NLP	Natural Language Processing
PCA	Principal Component Analysis
SPM	Statistical Process Monitoring
SPE	Squared Prediction Error
TE	Tennessee Eastman

Important notations and descriptions used in this work

**Notation**	**Description**
X	the process data
$x_{k}$	the kth observation variable
z	a latent variable of the observation variable
D	the dimension of the vector $x_{k}$
x	the observation variable
$\tilde{x}$	the reconstructed input
θ	the parameter of decoder network
ϕ	the parameter of encoder network
$x_{k}(\tau )$	on the τ time-shifted duplicate vectors of all the variables
${\tilde{x}}_{k}(\tau )$	a vector obtained by the flattening of the matrix $x_{k}(\tau )$
$g_{k}$	the input state (or new memory cell state) at k time
$c_{k}$	cell state (or final memory cell state) at k time
$h_{k}$	hidden state at k time
$i_{k}$	input gate
$f_{k}$	forget gate
$o_{k}$	output gate/update gate
σ	sigmoid function
$r_{k}$	reset gate
$n_{k}$	the new memory generated at k time
$x_{t}$	a new test sample
$z_{t}$	the latent representation for a new test sample
d	number of latent variable (d<D)
α	the significance level
f	the subnetwork
ς	the parameter of the subnetwork
$f_{\varsigma }^{ld}$	the latent subnetwork configured with parameter ς
$f_{\varsigma }^{rd}$	the residual subnetwork configured with parameter ς
$c_{ld}$	the cluster location center of the minimum volume of the hypersphere for latent projection
$R_{ld}$	the radius of the minimum volume of the hypersphere for latent projection
${\hat{x}}_{k}$	the residual for sample *k*
$c_{rd}$	the cluster location center of the minimum volume of the hypersphere for residual projection
$R_{rd}$	the radius of the minimum volume of the hypersphere for residual projection
$x_{t}^{i}$	the ith variable of the test sample
P	principal component projection matrix
$\tilde{P}$	residual projection matrix
ξ	the fault direction
g	the fault magnitude
${\hat{x}}_{t}$	the reconstructed vector
$\Xi_{t}$	a window-sized fault direction matrix
$G_{t}$	fault magnitude matrix
$\delta_{t}$	the negative reconstruction term
$T_{s}$	the sampling internal
$T_{\delta }$	the worst estimation time elapsed for the negative reconstruction term
$N_{thread}$	the required program thread number
